# The European Gaucher Alliance: a survey of member patient organisations’ activities, healthcare environments and concerns

**DOI:** 10.1186/s13023-014-0134-4

**Published:** 2014-09-02

**Authors:** Irena Žnidar, Tanya Collin-Histed, Pascal Niemeyer, Johanna Parkkinen, Anne-Grethe Lauridsen, Sandra Zariņa, Yossi Cohen, Jeremy Manuel

**Affiliations:** European Gaucher Alliance, Evesham House Business Centre, 48-52 Silver Street, Dursley, Gloucestershire GL11 4ND UK

**Keywords:** Gaucher disease, Patient organisations, Rare disease, Enzyme replacement therapy, Substrate reduction therapy

## Abstract

**Background:**

The European Gaucher Alliance (EGA) was established in 1994 and constituted in 2008 as an umbrella group supporting patient organisations for Gaucher disease. Every two years, the EGA conducts a questionnaire survey of member associations to help develop its priorities and annual work programme. Results of the latest survey are presented.

**Methods:**

Between June 2012 and April 2013, the 36 members and associate members of the EGA were asked to complete a questionnaire detailing membership numbers, disease specific treatments used by patients, means of access to treatment, availability of treatment centres and home infusions, sources of support for patients with Gaucher disease, patient organisations’ activities, collaborations, funding sources and any issues of concern. Questionnaires completed in 2012 were revised in January 2013 and responses analysed between July and September 2013.

**Results:**

Thirty three members returned data on one or more questions. Findings identified inequalities in access to treatment both within and between members’ countries. Three of 27 countries, for which data were available, relied totally on humanitarian aid for treatment and 6% of untreated patients in 20 countries were untreated because of funding issues, a situation many feared would worsen with deteriorating economic climates. Access to treatment and reimbursement represented 45% of members’ concerns, while 35% related to access to specialist treatment centres, home infusions and doctors with expertise in Gaucher disease. Member associations’ main activities centred on patient support (59% of responses) and raising awareness of Gaucher disease and patients’ needs amongst the medical community, government and healthcare decision makers and the general public (34% of responses). Twenty one (78% of respondents) indicated they were the only source of help for Gaucher disease patients in their country. For many, activities were constrained by funds; two members had no external funding source. Activities were maximised through collaboration with other patient organisations and umbrella organisations for rare diseases.

**Conclusion:**

The survey provided a ‘snapshot’ of the situation for patients and families affected by Gaucher disease, helping the EGA direct its activities into areas of greatest need.

## Introduction

The European Gaucher Alliance (EGA) (www.eurogaucher.org) was established in 1994 and constituted in 2008 as an umbrella group supporting patient organisations for Gaucher disease. It currently has 31 member associations in Europe (in 32 countries) and 8 associate members in 7 countries beyond Europe, which assist patients and families affected by Gaucher disease, a rare inherited metabolic disorder characterised by deficiency in the activity of the lysosomal enzyme glucocerebrosidase.

Deficiency in glucocerebrosidase results in the accumulation of the enzyme’s major substrate, glucocerebroside, in the lysosomes of macrophages. These substrate laden macrophages infiltrate the organs of the body leading to widespread and progressive disease involving, most commonly, the spleen, liver and bone, although the lungs, lymphatic system, kidneys, heart, skin, and central nervous system may also be involved [1,2]. Patients may exhibit signs and symptoms that include progressive enlargement of the spleen and liver with associated abdominal distension, pain and organ dysfunction; fatigue due to anaemia; gum and nosebleeds due to thrombocytopenia, coagulopathy and platelet dysfunction; increased susceptibility to infections as a result of neutropenia and impaired neutrophil function; neurological complications; and a wide range of skeletal manifestations such as bone pain, bone crises, bone remodelling abnormalities, osteopenia and osteoporosis, pathological fractures and osteonecrosis, which may lead to disability and the need for orthopaedic intervention [3,4]. Children, in addition, may exhibit growth retardation [5] and delayed pubertal development [6].

Type 1 Gaucher disease is by far the most common form of Gaucher disease affecting approximately one in 50,000 - 100,000 worldwide and one in 400 – 600 in the Ashkenazi Jewish population [7] and is traditionally defined by the absence of neurological symptoms, although a diagnosis of type 1 disease may not exclude the development of neurological symptoms later in life [8]. Type 1 Gaucher disease is highly heterogeneous in terms of age of onset, organs affected and rate of disease progression [3] and is also associated with an increased risk of cancer, especially haematological malignancies of B-cell origin, such as multiple myeloma [9] and an increased risk of Parkinson’s disease [10].

Neuronopathic forms of Gaucher disease affect approximately 6% of Gaucher disease patients. These include type 2 (acute neuronopathic) disease, where disease usually presents in the first 6 months of life, is rapidly progressive and frequently results in death before the age of two years, and type 3 (chronic neuronopathic disease) where neurological disease generally presents later in childhood and has slower progression than in type 2 disease [11].

Treatment for patients with non-neuronopathic symptoms first became available in 1991 in the form of enzyme replacement therapy (ERT), which aims to supplement deficient glucocerebrosidase by the intravenous administration of a functional glucocerebrosidase enzyme. The first enzyme to be used in the treatment of patients with Gaucher disease was mannose terminated human placental glucocerebrosidase, alglucerase (Ceredase®), which was replaced in 1994 by its recombinant form, imiglucerase (Cerezyme®), (both manufactured by Genzyme Corporation, Cambridge, MA, USA). A second ERT, velaglucerase alfa (VPRIV®; Shire Human Genetic Therapies, Dublin, Ireland) became available in 2010 and a third, taliglucerase alfa (Elelyso®; Protalix, Carmiel, Israel) has become available in the USA (approved by the FDA in 2012), Israel (in 2012) and Brazil (in 2013).

An alternative treatment approach, substrate reduction therapy (SRT) (miglustat [Zavesca®], Actelion Ltd, Basel, Switzerland) aims to reduce the accumulation of glucocerebroside by inhibiting its synthesis. This oral therapy was licensed in 2002 for mildly affected adult Gaucher patients for whom ERT is unsuitable [12]. An alternative SRT eliglustat tartrate (Cerdelga™; Genzyme Corporation, Cambridge, MA) for adult patients with type 1 Gaucher disease is currently available to patients as part of clinical trials [13]; decisions on marketing authorisation from the FDA and EMA for eliglustat tartrate are expected in 2014.

Treatments can transform patients’ lives by ameliorating many of the signs and symptoms of non neuronopathic disease [13–17], especially haematological and visceral aspects of disease, but they do not represent a cure for Gaucher disease; treatment is for life and interrupted treatment may incur a risk of clinical regression [18,19]. Skeletal complications may persist in some Gaucher disease patients despite treatment [20], and the impact of treatment on the incidence of associated comorbidities is not well understood [10,21]. Current treatments for Gaucher disease are also expensive (in the region, depending the patient’s weight and dosing regimen, of 200,000 – 380,000 US Dollars/154,000 – 292,600 Euros per patient per year), which creates significant challenges for health care providers. There is no currently available treatment that has any significant impact on the neurological manifestations of Gaucher disease [1]; the lack of treatment to address neuronopathic disease represents a major unmet need for affected patients.

As with many rare diseases, awareness of Gaucher disease amongst the medical community is low. Obtaining an accurate diagnosis can be a prolonged process involving many medical specialities and sometimes incurring unacceptable delays to appropriate medical care [22,23]. Individuals diagnosed with Gaucher disease may never have heard of their condition before and it is common for patients to have never met another affected individual. Patient organisations have an important role as a source of information and support for affected families; in raising awareness of the disease amongst the public and medical community, which can promote early diagnosis [24]; in defining and raising awareness of the specific issues that patients affected by disease face, and in facilitating research into the disease [25].

The principal roles of the EGA in supporting patient organisations for Gaucher disease are to provide guidance and encouragement to these groups, to communicate the latest developments in understanding Gaucher disease; to encourage research into Gaucher disease; to work with healthcare professionals and scientists to define priorities in the understanding of Gaucher disease and its management; to represent the interests of Gaucher patients to European and international organisations and bodies; and to help ensure that appropriate treatment is available to all Gaucher disease patients who need it.

Healthcare professionals and scientists play an important role in supporting the work of patient groups and have involved patient group representatives in scientific meetings about Gaucher disease. Patient representatives were invited to join the first meeting of European Working Group on Gaucher disease (EWGGD) in 1994, and have continued to take part in these meetings, which are held every two years. During these events, EGA patient representatives take time to discuss their own priorities and concerns. As part of activities to prepare for these meetings and define how to better support its members and develop its work programme, the EGA conducts a questionnaire survey of its member associations every two years to identify common areas of interest, priorities and concerns for patient organisations and the Gaucher disease patients they represent. The results of the latest survey prepared for the 2014 EWGGD meeting in Haifa, Israel, are outlined here.

## Methods

Between June 2012 and April 2013, the 36 members and associate members of the EGA (in Austria, Belgium, Bosnia & Herzegovina, Bulgaria, Canada, Czech Republic, Denmark, Estonia, Finland, France, Germany, Greece, India, Israel, Italy, Jordan, Latvia, Lithuania, Macedonia, Mexico (2 organisations), the Netherlands, Norway, Poland, Romania, Russia, Serbia, Slovakia, Slovenia, South Africa, Spain, Sweden, Switzerland, United Kingdom [UK] & Republic of Ireland (RoI) (1 organisation), Ukraine and United States of America [USA]) were asked to complete a questionnaire detailing:

Association membership:

- Membership numbers and the proportion of members that are patients with Gaucher disease and/or family members
- The approximate number of all Gaucher patients in the member organisation’s country (and if known, the number of children under 18 and the number of adults)

Treatment:

- Approximate numbers of patients receiving treatment (and the treatment type)
- Approximate numbers of patients on a humanitarian aid programme
- Approximate numbers of patients not receiving treatment (and reasons, if known)

Healthcare:

- The names of main hospitals where Gaucher patients may be treated
- Whether home infusions are available in the member’s country

The patient organisation’s activities:

- The activities that the patient organisation offers
- Membership of other national/international organisations
- Whether the patient association works with any other organisations nationally on a regular basis
- How the patient association is funded
- Whether there is any other means of support for patients with Gaucher disease in the member’s country
- Any concerns that the patient organisation or its members face in the member country

The questionnaires were completed between June 2012 and April 2013. Those completed in 2012 were revised in January 2013. Responses were analysed between July and September 2013.

## Consent

This survey was carried out with the voluntary participation of EGA member organisations. All member organisations’ representatives reviewed and agreed the final manuscript before submission for publication. The study questionnaire, which was prepared by and approved by the EGA Board of Directors, did not ask for any identifiable personal details about participating organisations’ members or any identifiable details of their clinical history, which required the consent of any specific individual. The questionnaire and conduct of the survey were compliant with the principles of the Declaration of Helsinki.

## Results

Of the EGA’s 36 member and associate member patient organisations, 33 member organisations returned data on one or more questions (Table 1). Of these 33 member associations, 23 were specific to Gaucher disease (in Austria, Bulgaria, Canada, Czech Republic, Denmark, Estonia, Finland, German, Israel, Italy, Jordan, Lithuania, Mexico [AGdM], Norway, Poland, Russia, Serbia, Slovenia, Spain, Sweden, UK & RoI, Ukraine and USA); ten associations had a wider remit and included, for example, other lysosomal storage diseases (LSDs), other rare diseases or metabolic diseases (Belgium, Bosnia & Herzegovina, Greece, Latvia, Macedonia, Mexico [PPuDM], the Netherlands, Romania, South Africa and Switzerland). Three patient associations (from Croatia, Paraguay and Moldova), which joined the EGA between June and September 2013, are not included in the survey.

**Table 1:** **Contributing EGA member organisations**

**Country**	**Organisation**	**Scope**
Austria	Österreichische Gaucher Gesellschaft/Austrian Gauchers Association	Gaucher disease
Belgium	Belgische Organisatie voor Kinderen en Volwassesn met en Stofwisselingsziekte/Belgian Association for Metabolic Diseases	Metabolic diseases
Bosnia & Herzegovina	Udruženje oboljelih od rijetkih bolesti u Bosni i Hercegovini/Association of people with rare diseases in Bosnia & Herzegovina	Rare diseases
Bulgaria	Национална Асоциация на Болните от Гоше/National Association of Gaucher patients in Bulgaria	Gaucher disease
Canada	National Gaucher Foundation of Canada	Gaucher disease
Czech Republic	Občanské sdružení rodičů dětí a dospělých postižených Gaucherovou chorobou/Czech Gaucher Association	Gaucher disease
Denmark	Gaucher Foreningen i Danmark/Gaucher Association Denmark	Gaucher disease
Estonia	Gaucher Eesti MTÜ/Estonian Gaucher Association	Gaucher disease
Finland	Suomen Gaucher Yhdistys/Finnish Gaucher Association	Gaucher disease
Germany	Gaucher Gesellschaft Deutschland/German Gaucher Association	Gaucher disease
Greece	Πανελλήνιος Σύλλογος Ασθενών & Φίλων Πασχόντων από λυσοσωμικά Νοσήματα ‘Η Αλληλεγγύη’/Patients and friends of those suffering from lysosomal storage disorders ‘The Solidarity’	LSDs
Israel	Israeli Gaucher Association/העמותה הישראלית לגושה	Gaucher disease
Italy	Associazione Italiana Gaucher/Italian Gaucher Association	Gaucher disease
Jordan	Jordanian Gaucher Association/جم ع ية ال ع ناي ة ب مر ضى غو ش يه ال خ يري ة الأردن ية	Gaucher disease
Latvia	Reto slimību biedrība “Caladrius”/Society of Rare Diseases Caladrius	Rare diseases
Lithuania	Lietuvos Gošė Asociacija/Lithuania Gaucher Association	Gaucher disease
Macedonia	Здружение на граѓани за ретки болести “Живот со Предизвици” – Битола/Association of citizens for rare diseases - Life with Challenges	Rare diseases
Mexico	Asociacion Gaucher de Mexico/Gaucher Association of Mexico	Gaucher disease
Mexico	Proyecto Pide un Deseo México, i.a.p./Make a Wish Project	LSDs
The Netherlands	VKS – Diagnosegroep Gaucher/Gaucher Diagnosis Group	Metabolic diseases
Norway	Gaucher Foreningen Norge/Gaucher Association Norway	Gaucher disease
Poland	Stowarzyszenie Rodzin z Chorobą Gauchera/Association of Families with Gaucher disease	Gaucher disease
Romania	Fundatia Romana Pentru Bolile Lizozomale/Romanian Foundation for Lysosomal Diseases	LSDs
Russia	Межрегиональная общественная организация «Содействие инвалидам с детства, страдающим болезнью Гоше и их семьям»/Interregional non-government organization “Assistance to invalids suffering from Gaucher disease and their families”	Gaucher disease
Serbia	Udruženje gradjana za pomoć u lečenju obolelih od Gošeove bolesti (UGOŠ)/Association for help in treatment of patients suffering from Gaucher’s disease	Gaucher disease
Slovenia	Društvo bolnikov z gaucherjevo boleznijo Slovenije/Slovenian Gaucher Association	Gaucher disease
South Africa	The Gaucher and LSD Society of South Africa	Gaucher disease/LSDs
Spain	Asociación Española de Enfermos y Familiares de la Enfermedad de Gaucher/Spanish association of Gaucher patients and their relatives	Gaucher disease
Sweden	Morbus Gaucherföreningen i Sverige/Swedish Gaucher Disease Association	Gaucher disease
Switzerland	Vaincre les Maladies Lysosomales – Suisse/Swiss Association for Overcoming Lysosomal Storage Disorders	LSDs
UK & Republic of Ireland (RoI)	The Gauchers Association	Gaucher disease
Ukraine	Всеукраїнська громадська організація “Об”єднання інвалідів-хворих на хворобу Гоше/All-Ukrainian Gaucher Association	Gaucher disease
USA	National Gaucher Foundation	Gaucher disease

### Association membership

Of the 33 contributing patient associations, 28 (85%) completed data on membership numbers, which totalled approximately 3322 individuals (data not shown). Of the 28 contributing groups, 17 (61%) provided information on the numbers of Gaucher patients in their membership (as distinct from patients with other disorders, unaffected relatives or other members). Of an approximate total 1771 members in these 17 associations, approximately 918 (52%) were patients with Gaucher disease. Of the 17 contributing groups, 13 (76%) were Gaucher disease specific associations. Gaucher disease patients represented 69% of overall membership in these organisations (1204 members and 825 Gaucher disease patients) (Table 2).

**Table 2:** Association membership*

**Member association’s country**	**Total membership**	**Total GD patient members**	**GD patients as** **%** **of membership**	**Total GD patients in country**	**No. of GD children (under 18) in country**	**No. of GD adults in country**	**GD patients in association as** ** % ** **of patients in country**
Austria	40	20	50	20	2	18	100
*Bosnia & Herzegovina*	*237*	*4*	*2*	*4*	*0*	*4*	*100*
Denmark	32	17	53	17	5	12	100
Estonia	4	3	75	4	0	4	75
Finland	15	4	27	10	2	8	40
Germany	210	155	74	335	35	300	46
*Greece*	*200*	*79*	*40*	*80*	*10*	*70*	*99*
Israel	500	350	70	850	300	550	41
Italy	114	114	100	240	0	240	48
Jordan	30	10	33	28	19	9	36
*Latvia*	*30*	0**	*0*	*3*	*1*	*2*	*0*
Lithuania	9	7	78	8	2	6	88
*Macedonia*	*100*	*10*	*10*	*12*	*6*	*6*	*83*
Serbia	35	28	80	36	7	29	78
Slovenia	37	17	46	19	2	17	89
Spain	110	60	55	300	100	200	20
Sweden	68	40	59	55	10	45	73
**TOTAL**	**1771**	**918**	**52**	**2021**	**501**	**1520**	**45**

GD: Gaucher disease; *Only member organisations providing complete data (17 respondents); **The parents of child with GD are members; child is not a member; Italics: Associations for GD + other diseases. Not in italics: GD specific associations (n = 13).

GD patients represented 52% of overall membership in responding patient organisations (918 GD patients of 1771 total members).

GD patients represented 69% of overall membership in GD specific organisations (825 GD patients of 1204 total members).

The total number of Gaucher patients (in the 17 member associations that provided sufficient data) represented 45% of the estimated total numbers of Gaucher disease patients in all their countries at the time of data collection (Table 2). Percentages for individual countries ranged from 100% (in Austria, Bosnia & Herzegovina, and Denmark) to zero (in Latvia where parents of a child with Gaucher disease were members of the patient association, but their child was not). The median value for Gaucher disease patient membership was 75%.

### Treatment

Twenty seven member organisations contributed data on treatments received by a total of 2043 patients in their countries (not necessarily all members of contributing member associations). Of these, 1317 (64%) were treated with imiglucerase ERT, 466 (23%) received velaglucerase alfa ERT, and 86 (4%) received taliglucerase alfa ERT; 75 (4%) received miglustat SRT, 93 (5%) received eliglustat tartrate SRT, and 6 (<1%) received other treatments, such as alglucerase or an investigational therapy in early stage clinical trials (Table 3).

**Table 3:** **Numbers of patients receiving Gaucher disease specific treatments**

**Member association’s country**	**Imiglucerase**	**Velaglucerase alfa**	**Eliglustat tartrate**	**Miglustat**	**Taliglucerase alfa**	Other*	**Total**
Austria	11	5	4	1			21
Belgium	22	3		1			26
Bosnia & Herzegovina	3	1	0				4
Bulgaria	13						13
Canada	60	13	13	11	2		99
Czech Republic	21	6		2			29
Denmark	11	5					16
Estonia	3						3
Finland	6	2		1			9
Germany	230	61	9		1		301
Greece	57	6	3	6			72
Israel	80	160	10		60	5	315
Italy	113	35		10			158
Jordan	16	1					17
Latvia	2						2
Lithuania	8						8
Macedonia	4						4
Norway	5	8					13
Romania	55	1	2				58
Russia	258	6	40				304
Serbia	13		6		8		27
Slovenia	13	4					17
South Africa	48			3	5		56
Spain	80	50	4	30	5		169
Sweden	37	6		3			46
UK & RoI	112	93	2	7	5	1	220
Ukraine	36						36
**TOTAL**	**1317**	**466**	**93**	**75**	**86**	**6**	**2043**
**% of total**	**64**	**23**	**5**	**4**	**4**	**0.3**	

*Other treatments: Israel: oral enzyme replacement therapy (PRX-112) in clinical trial [Protalix, Carmiel, Israel], UK & RoI: 1 alglucerase.

A total of 66 patients received treatment as part of a humanitarian aid programme; 20 (30%) were from Ukraine (56% of all patients receiving treatment in the country), 13 (20%) from Serbia (48% of treated patients in the country), 16 (24%) from Jordan (100% of treated patients), 5 (8%) from South Africa (9% of treated patients), 4 (6%) from Macedonia (100% of treated patients), 4 (6%) from Bosnia & Herzegovina (100% of treated patients), and 1 (2%) from Latvia (50% of treated patients). In summary, 100% of patients received their treatment through humanitarian aid in 3 countries; 40 - 60% of patients received treatment through humanitarian aid in four countries and less than 10% of patients received treatment though humanitarian aid in one country (Table 4).

**Table 4:** **Number of Gaucher disease patients receiving treatment as part of a humanitarian aid programme**

**Member association’s country**	**No. on HA**	**Treated patients in country**	**% of treated in country**
Bosnia & Herzegovina	4	4	100
Jordan	16	16	100
Latvia	1	2	50
Lithuania	3	8	38
Macedonia	4	4	100
Serbia	13	27	48
South Africa	5	56	9
Ukraine	20	36	56
**Total**	**66**	**153**	**43**

Twenty associations returned information on untreated patients. These totalled 720 individuals, 611 (85%) of whom were untreated because their clinical condition did not warrant or qualify for treatment; 46 (6%) were untreated because of lack of funding; 50 (7%) for whom no specific reasons were cited, and 13 (2%) who were untreated through personal choice (Table 5).

**Table 5:** **Reasons why some patients were untreated**

**Member association’s country**	**No. untreated**	**Patient choice**	**Clinical reasons**	**Funding issues**	**No specific reason given**
Bulgaria	3	3			
Canada	70		70		
Czech Republic	6				6
Denmark	1		1		
Estonia	1				1
Finland	1	1			
Germany	1	1			
Israel	535		535		
Jordan	15			15	
Latvia	1		1		
Macedonia	5			5	
Norway	1		1		
Russia	19				19
Serbia	9	3		6	
Slovenia	2				2
South Africa	20				20
Spain	5	3			2
Sweden	3		3		
Switzerland	2	2			
Ukraine	20			20	
**Total**	**720**	**13**	**611**	**46**	**50**
**% of total**		**2**	**85**	**6**	**7**

### Healthcare

Thirty one patient groups provided information on the number of main hospitals where patients with Gaucher disease are treated (Table 6). Overall, no main treatment centres were specified by 2 of 31 (6%) respondents (from Lithuania and Switzerland). In Lithuania, patients were treated in local hospitals and in Switzerland it was stated that patients can be treated anywhere as long as providers can provide evidence that they have been adequately trained to administer treatment (trained in preparation, intravenous infusion care and anaphylactic shock management). Five respondents (16%) reported one main treatment hospital; 14 (45%) reported 2–5 main hospitals, 6 (19%) have 6–10 main treatment hospitals and 4 (13%) have 11 or more main hospitals. Thirty two member associations reported on the availability of home infusions for enzyme replacement therapy. Home infusions were stated to be available in 20 of 33 (61%) countries (RoI counted separately in this case as policy on home infusion differs between the UK and RoI) (Table 6).

**Table 6:** **Number of main hospitals where Gaucher patients are treated and availability of home infusions**

**Number of main hospitals**	**Member association’s country**	**Home infusions available**	**Home infusions not available**
None	Lithuania, Switzerland	Switzerland	Lithuania
1 main hospital	Czech Republic, Estonia, Macedonia, the Netherlands, Ukraine	The Netherlands	Czech Republic, Estonia, Macedonia, Ukraine
2-5 main hospitals	Bosnia & Herzegovina, Bulgaria, Denmark, Finland, Israel, Jordan, Latvia, Norway, Russia, Serbia, Slovenia, South Africa, Spain, Sweden	Bosnia & Herzegovina, Bulgaria, Denmark, Finland, Israel, Norway, Russia, Slovenia, South Africa, Spain, Sweden	Jordan, Latvia, Serbia
6-10 main hospitals	Belgium, Germany, Greece, Italy, Romania, UK & RoI	Germany, Italy, Romania, UK	Belgium, Greece, RoI
11 or more main hospitals	Austria, Canada, Poland, USA	Austria, Canada, USA	Mexico, Poland
31 respondents		32 respondents covering 33 countries (UK and RoI counted separately as policy on home infusion differs)	

### The patient organisations’ activities

**Figure 1:**
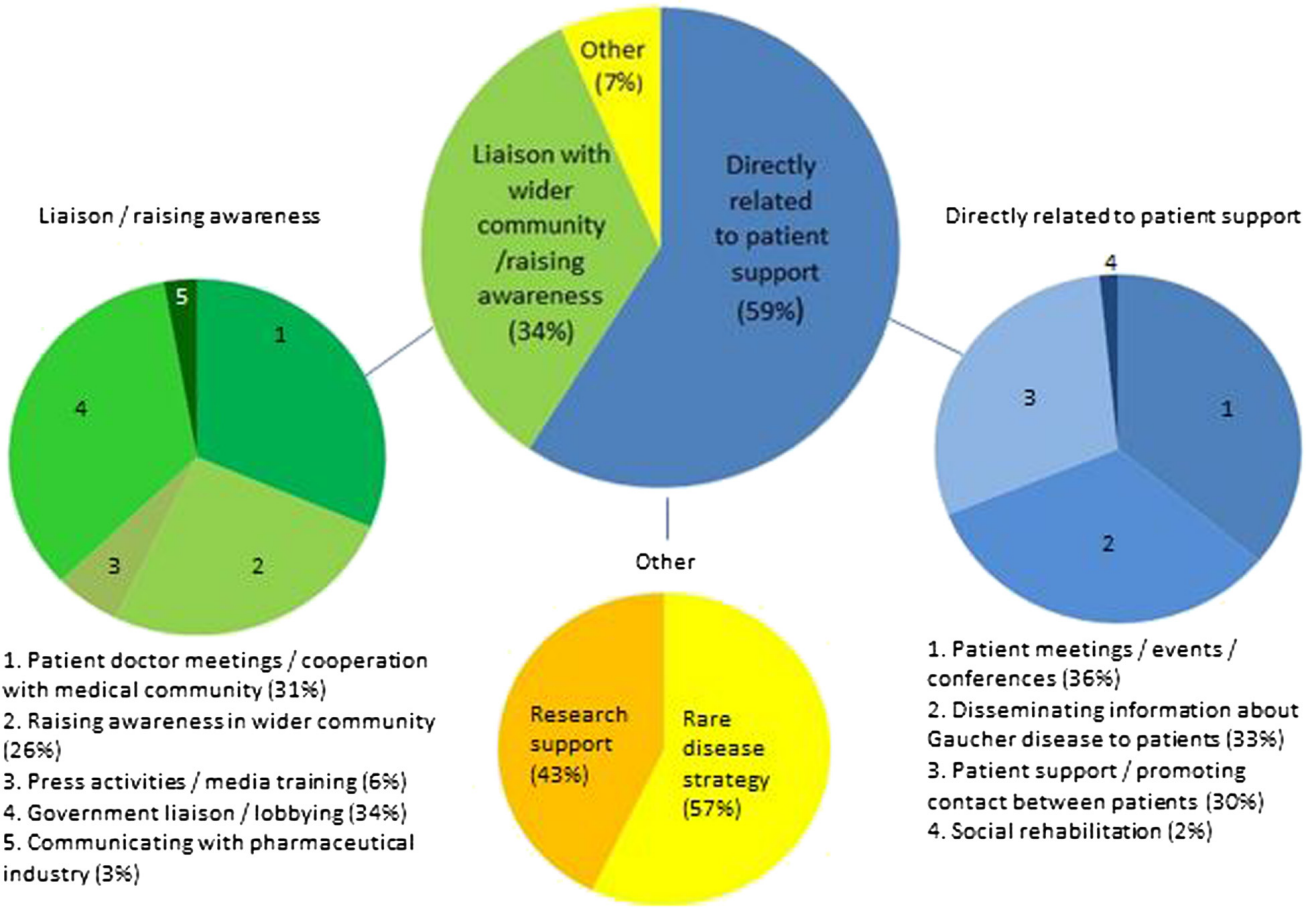
**Member associations’ activities.**

Thirty one associations provided 103 responses to describe their activities. The responses were categorised as shown in Figure 1. The activities undertaken by organisations, varied considerably; 59% of all activities were directly related to providing support to patients and promoting contact between patients, organising events for patients, disseminating information to Gaucher patients and families about the disease and providing help with social rehabilitation, 34% of activities were related to raising awareness of Gaucher disease and the issues that patients face amongst the medical community, the wider (non-Gaucher) community, with government, and with the pharmaceutical industry; 7% of activities were related to rare disease strategy and research into Gaucher disease.

### Other sources of support for Gaucher patients

Of 27 members who responded, 21 (78%) indicated that they were the only source of support for Gaucher patients in their countries (Belgium, Canada, Denmark, Germany, Greece, Israel, Italy, Jordan, Latvia, Lithuania, Macedonia, the Netherlands, Norway, Poland, Romania, Russia, Serbia, Slovenia, Sweden, UK & RoI, and Ukraine). Six member associations (22%) indicated other sources of support for patients. These included support available through rare disease organisations (Austria, Bulgaria, Mexico, South Africa, Spain, and USA) and two (Spain, and the USA) referred to other Gaucher disease specific patient organisations, such as the Spanish Federation of Gaucher patients and, in the USA, the Children’s Gaucher Research Fund, an organisation supporting research into type 2 and type 3 Gaucher disease, but which may also help families by putting them in touch with each other. The member from the USA also mentioned that there was a financial assistance programme available to Gaucher patients.

### Membership of other national/international organisations

Twenty five member associations completed the question on membership of other national/international organisations. Of these 25 associations: 18 (72% of organisations) were members of national or international rare disease organisations (such as EURORDIS, NORD); 1 (4%) was a member of another Gaucher disease-related organisation (excluding the EGA), 6 (24%) were members of other patient organisations (not Gaucher disease related) and 2 (10%) were members of other national or international bodies, such as a health care coalition and a genetic counselling organisation. Five (20%) did not belong to any other organisation.

### Work with nationally

Twenty eight members responded to the question about whether they worked with any other organisations nationally on a regular basis: 5 associations (18%) (Latvia, Lithuania, Mexico AGdM, Poland, Ukraine) did not work regularly with others, although some worked with other organisations on an occasional basis. Latvia, for example, reported a collaboration with the Latvian Haemophilia Association for Rare Disease Day and Lithuania, as a relatively new society, has yet to establish regular working relationships; 23 members (82%) (from Austria, Belgium, Bosnia & Herzegovina, Canada, Denmark, Finland, Germany, Greece, Italy, Jordan, Macedonia, Mexico PPuDM, the Netherlands, Romania, Russia, Serbia, Slovenia, South Africa, Spain, Sweden, Switzerland, UK & RoI, and USA) provided information on their, often multiple, collaborations. Fifteen of the responding 28 member organisations (54%) indicated that they collaborated with pharmaceutical companies, 12 (43%) worked with rare disease organisations, 11 (40%) worked with other patient organisations, 6 (21%) worked with treatment centres and the medical community; 4 (14%) worked with other organisations such as the media, non-pharmaceutical companies, and youth councils, and 3 (11%) worked with government.

### Funding

Thirty members provided information about sources of funding for their patient organisations. Two (7%) (Jordan and Ukraine) reported that they had no source of external funds. Eighteen of the respondents (60%) cited a variety of subsidies, grants and miscellaneous donations that included, for example, lottery money (Denmark), donations from health insurance fund (Germany), income tax subsidies (Lithuania, Romania), a governmental small subsidy for volunteers helping in the patient organisation and subsidies from various cities (Belgium) and grants and donations from individuals (Mexico PPuDM) and organisations (Bosnia & Herzegovina, Canada, Greece, Latvia, Serbia, Slovenia, Switzerland, USA). Pharmaceutical companies provided support to 15 patient organisations (50% of respondents), either on an annual basis and/or in response to requests for unrestricted grants for specific meetings and events. Six member associations (20%) raised funds through membership fees for patients and families (Belgium, Germany, Poland, Serbia, Spain, Switzerland) and six (20%) held fund raising events such as sponsored runs, family days, concerts and family events (Belgium, Latvia, the Netherlands, South Africa, UK & RoI, and the USA).

### Concerns expressed by patient organisations

Twenty seven members outlined the issues their association and Gaucher patients face. Fifty five points of concern were raised. Of these 25 (45%) related to access to Gaucher specific treatment, 19 (35%) were related to the medical care received by patients; 5 (9%) related to the difficulties faced by Gaucher patients in their everyday lives, and 6 (11%) were issues related to the management and future of patient organisations (Table 7).

**Table 7:** **Concerns of member associations**

**Concern**	**No. of responses**	**Member association’s country**
**Medical care (Total 19 responses from 12 member associations)**		
Lack of information/limited knowledge among healthcare professionals/lack of Gaucher disease specialists	7	Finland, Latvia, Lithuania, Macedonia, Mexico PPuDm, Slovenia, Switzerland
Lack of consensus on management approach for Gaucher disease	2	Slovenia, Switzerland
Unmet needs: neuronopathic Gaucher disease, bone, mental health services/psycho-social support for patients	3	Slovenia, UK & RoI, USA
Lack of information on new therapies	1	Germany
Need for home infusions	1	Mexico PPuDM
Need for diagnostic centre/issues with delayed diagnosis/finding undiagnosed patients, need for specialist centre	4	Mexico AGdM, Slovenia, USA, Russia
Lack of a national Gaucher disease registry	1	Slovenia
**Access to treatment (Total 25 responses from 20 associations)**		
Treatment access/inequalities in access	9	Bosnia & Herzegovina, Canada, Greece, Jordan, Latvia, Mexico PPuDM, Serbia, South Africa
Low treatment doses and delayed time to treatment	3	Norway, Romania, Spain
Imiglucerase shortage/way shortage was managed	4	Italy, Poland, South Africa, Spain
Changing economic environments/concerns for future reimbursement/specialised services	7	Denmark, the Netherlands, Spain, Sweden, Switzerland, UK & RoI, USA
Lack of collaboration with pharmaceutical companies regarding humanitarian aid	1	Jordan
Lack of dialogue with administrative authorities/hospitals/payers	1	Poland
**Living with Gaucher disease (Total 5 responses from 5 associations)**		
Difficulties for Gaucher disease patients in finding jobs	1	Bosnia & Herzegovina
Patients’ fear of repercussions if disease is known for insurance/barrier to diagnosis	1	USA
Obtaining disability status for patients	1	Russia
Lack of information on Gaucher disease for the general public	2	Macedonia, Switzerland
**Patient organisations (Total 6 responses from 5 associations)**		
Lack of Gaucher disease patient interest in patient organisation	3	Belgium, Germany, Slovenia
Need to collaborate (between patient organisations)	1	Mexico PPuDM
Changing financial situation and lack of funds to support patients	1	USA
Lack of umbrella organisation for rare diseases	1	Slovenia

A total of 27 member associations responded (55 responses).

## Discussion

This survey provides a ‘snapshot’ of the activities, interests and issues of concern of 33 EGA member associations between January 2013 and April 2013.

Patients with Gaucher disease are relatively fortunate amongst sufferers of rare diseases in that there are multiple disease-specific treatments available to them able to address some of the non neuronopathic aspects of disease. Data from 27 member associations suggested that the longest established treatment, imiglucerase ERT, was by far the most widely used treatment (64% of treated patients) followed by velaglucerase alfa ERT (23%), eliglustat tartrate SRT (5%), taliglucerase alfa ERT (4%), and miglustat SRT (4%) (Table 3). Some patients, however, remain untreated. For 720 patients for whom a reason for lack of treatment was known, approximately 85% (611 patients) were untreated because of clinical reasons, i.e. that their disease did not warrant treatment. Eighty eight per cent of these individuals came from Israel where Gaucher disease is relatively common [26] and where there is a high frequency of the N370S/N370S genotype, which is associated with a milder, later onset phenotype (although not exclusively) [27]. Forty six patients (6%) were reported to be untreated because of funding issues (Table 5).

Access to treatment accounted for almost 50% of concerns raised by member organisations and highlighted inequalities both within and between countries. Canada’s 10 provinces and three territories, for example, each have different health care policies and depending on where patients live, they may or not have access to reimbursement for treatment. Respondents reported that there is no funding for the treatment of Gaucher disease in Jordan, and no funding for adult patients in Latvia and Ukraine. While there is a programme for funding treatments for rare diseases in Macedonia, none of Macedonia’s four treated Gaucher patients received funding through this programme. All treated Gaucher disease patients in Bosnia & Herzegovina, Jordan, and Macedonia were reliant on humanitarian aid (Table 4). Concerns were raised that worsening economies in many countries might result in pharmaceutical companies being unable to continue supporting humanitarian aid programmes to the same extent.

Even where treatment for Gaucher disease is available through national and private funding schemes, fears were expressed that access to treatment might not be maintained in the future for budgetary or other reasons. The Swiss Gaucher patients association cited the case of the withdrawal of reimbursement by all insurance companies in Switzerland for late-onset Pompe disease patients regardless of their clinical condition as a result of a reassessment of the therapy’s clinical efficacy [28]. While the respondent pointed out that treatment for Gaucher disease has a stronger evidence base regarding outcomes, it highlights inequality of access to treatment in different countries for rare disease and demonstrated how patients’ treatment options can be suddenly limited as a result of changing healthcare policies. Further examples include the withdrawal of the exemption for Gaucher disease from cost effectiveness assessment in Sweden due to high price, and discussions in the Netherlands on whether treatment for Fabry disease and Pompe disease should be fully reimbursed [29].

Concerns for the on-going availability of treatments and the funding of new treatment for rare diseases have also been raised in the UK following the transfer of the assessment for medicines for very rare disease from the Advisory Group for National Specialised Services (AGNSS) to the National Institute for Health and Clinical Excellence (NICE) in April 2013. Fears have been expressed that NICE may struggle to reconcile the high cost of some treatments for very rare disease with its funding thresholds [30]. A recent study using the UK as a model, presents compelling legal arguments (involving disability legislation, national and organisational constitutions, judicial review, tort law and human rights legislation) for the continued provision of treatment for orphan diseases despite economic pressure to reduce funding, and which may be applicable across the EU in negotiating national reimbursement guidelines [29].

Several members indicated that budget constraints were making treatment harder to obtain incurring delays in treatment and treatment initiation, and resulting in treatment at low doses even in patients with established disease (Norway, Romania, Spain). Treatment at lower doses was also, in part, a persisting consequence of the recent shortage in imiglucerase and the consequent sharing of available enzyme supplies amongst patients [31]. Although patients in some countries were able to switch to an alternative therapy to maintain treatment, others were unable to do so because alternative treatments had not been approved for marketing in their country. As requests for marketing authorisations have to be initiated by pharmaceutical companies it was felt that there might not be sufficient financial incentive for companies to enter the market in countries with few patients. The concern was also expressed that low dose treatment may become the norm regardless of drug supplies. The question of optimal enzyme dose regimens for Gaucher patients is a topic of discussion [32–34] but it has been recommended by clinical experts in Gaucher disease, that treatment should be tailored to patient’s needs [35]. Dose decisions, therefore, should be the domain of the treating clinicians, and not dictated by inflexible administrative policies. While initial reports during the imiglucerase shortage tend to suggest that drug reduction did not induce substantial changes in clinical laboratory results, it did appear to influence feelings of well-being of some Gaucher patients [36]. As reported by the Italian respondent, many patients were reluctant to switch to new therapies because their doctors were reticent and had limited knowledge of alternative therapies, which may have influenced patients’ perceptions.

Respondents also raised concern over the way that the imiglucerase shortage was managed in some regions and that there were vast differences in practice amongst treating hospitals. Anecdotal reports from respondents suggested that treatment had been withheld during the shortage in some centres in some countries despite the availability of low dose treatment for all, or administered at even lower doses than had been made available for patients. In contrast, the shortage was reported to have been managed well in other centres with enzyme supplies being allocated to patients based on a rational assessment of each patient’s clinical need by the doctor in charge.

As well as differences in access to treatment for Gaucher disease, standards of healthcare for Gaucher patients in respondents’ countries varied (accounting for 35% of responses on concerns). In common with a previous report on healthcare for patients with rare disease, Gaucher patients may have problems accessing good quality health care services [24]. Seven member associations’ representatives (26% of respondents) felt that knowledge about Gaucher disease amongst the general medical community in their country was inadequate (Table 7) making it difficult for patients to obtain information about their disease. This lack of awareness and lack of knowledge may result in delayed diagnosis as reported earlier [22]. Four member associations referred to a lack of Gaucher disease expertise in their countries (Finland, Mexico, Lithuania, Slovenia). For example, in Lithuania, it was reported that there was only one doctor (a paediatrician) with specialist knowledge of Gaucher disease, and that this doctor could not be accessed by adults through the healthcare system, and in Slovenia, there were no rare disease reference centres and a lack of specialists, such as radiologists, well experienced in Gaucher disease.

Although the questionnaire survey identified the number of main hospital where Gaucher patients may be treated in member association countries, it was not clear in all cases, which ones were designated, or recognised, centres of expertise or whether the main hospitals offered specialist expertise in the management of Gaucher disease. As reported by the Swiss respondent, not all main hospitals have a Gaucher disease specialist on site. While a number of designation criteria for national centres of expertise in rare disease in the European Union were defined in 2006 [37] and quality criteria defined in 2011 [38], designation criteria for centres of expertise vary between countries and sometimes between regions and may depend on the county’s policy for rare disease. Only a few member states have officially designated centres of expertise for rare diseases financed by the relevant health authorities (Denmark, Norway, Spain and the UK). Italy has regionally designated centres. Some countries have non-designated centres of expertise for rare diseases, which may be acknowledged by some authorities, while some are recognised only by reputation, and some are self-declared centres of expertise [39]. A number of European countries plan to elaborate designation procedures for centres of expertise for rare diseases in the future, mostly within the scope of a national plan/strategy for rare diseases. The European Commission recommended, that all member states adopt a national plan or strategy for rare disease by 2013 [40].

Home infusions were not an option for patients living in 13 countries (Table 6). While it might be expected that home infusion would be available in countries with restricted numbers of hospitals where patients may receive treatment for Gaucher disease, this did not appear to be the case (Table 6). While not all patients may feel comfortable with the concept of receiving treatment away from a medical environment, time spent travelling to treatment centres can have an impact on work, education, finances and quality of life [41,42]. Home treatment may offer a more convenient alternative and for some may represent the only practical way of receiving treatment when distances to treatment centres are great. It was reported from Serbia, for example, that two patients receiving treatment through humanitarian aid at their local hospital had to stop treatment because the donor pharmaceutical company would no longer allow infusions in the hospital (reason unspecified). Home infusions although once available were no longer offered. In order to continue treatment the patients had to take time away from work every fortnight to travel to Serbia’s treatment centre in Belgrade. As well as the time, cost and inconvenience involved, absence from work on a regular basis may mean that patients have to inform employers of their health status and, as the Serbian respondent reported, many may not wish to disclose this information.

Three respondents highlighted the lack of adequate mental health provision for Gaucher disease patients. As with any patient suffering from a chronic medical condition, patients with Gaucher disease may experience psycho-social complications [43]. Specific concerns for Gaucher disease patients include a difficulty coping with diagnosis, dealing with the effects of pain and fatigue on job, career, and recreational activities, difficulties with social life, emotional problems such as depression, and psychological distress [42].

Over 80% of responding member associations indicated that they were the only source of help for Gaucher patients and their families in their country. The main activities of member associations were centred on patient support (59% of responses), which included helping individual patients, promoting and facilitating contact between patients in similar situations, holding patient and family meetings, events and workshops, and providing information on Gaucher disease and its treatment and other services, such as help with social rehabilitation, work, and benefits (for example, disability benefit) where available. Several associations reported producing and disseminating information about Gaucher disease and new developments relating to Gaucher disease through websites, newsletters and other publications, such as leaflets and booklets. Activities also included guiding patients through any concerns associated with treatment, helping patients in their interaction with doctors, medical and administrative authorities, advising individuals on where to go to for diagnosis if they suspected Gaucher disease, help with hospital admissions, help in obtaining access to treatment and helping patients learn about the health economic system in their country.

Over one third of patient association activities related to raising awareness of Gaucher disease in the medical community or amongst the general public (including government and health care decision makers). Twelve member associations reported an active role in cooperating with government to raise awareness and educate about rare disease, to inform government of specific needs and gaps in the law for patients with a rare disease, to defend the right of patients with rare disease and to negotiate with government and insurance funders for treatment reimbursement. Ukraine for example, has been active in lobbying the President of Ukraine, the Supreme Council of Ukraine, the Health Protection Ministry, to request an increase public financing for children with rare diseases and to develop the programme of public financing for adult patients; and Bosnia & Herzegovina, Latvia, Macedonia, and Poland reported participating actively in the development of national plans for rare diseases.

One specific problem identified by the respondent from Macedonia was the lack of educational materials in the local language, which severely hampered increasing awareness of Gaucher disease. From the patient perspective it meant that patients had no materials to give to members of the medical profession, to employers, teachers or members of their own family who might need to be informed about Gaucher disease. Similarly, lack of written material made it difficult to deal with government and to raise awareness among the general public.

The scale of activities undertaken by each member organisation are inevitably influenced by the financial resources available to them but even the two member patient organisation without external funding resources (Jordan and Ukraine) were able to provide support and information to patients and provide a point of contact for patients and families wishing to talk to each other, to lobby government for increased funding for patients with rare diseases (Ukraine) and to raise awareness of rare diseases, help in obtaining a diagnoses for people affected by Gaucher disease, and work with hospital and doctors (Jordan). Although not explicitly stated by all, these activities rely on the willingness of association organisers to donate their time, and often their personal money, to support any expenditure.

The single greatest specified external source of funding was the pharmaceutical industry, although some associations exploited other resources such as lottery funds, subsidies supported by income tax refunds (gift aid schemes), and unspecified donations. Some associations charged membership fees and funds were also raised through fund raising events, which can help source funds from outside the organisation. The respondent from the USA pointed out that funding has become more difficult ‘because of the economy and cutbacks from the pharmaceutical industry’. Without proper funding, it was stated that programmes may have to be reduced.

To help maximise financial resources and to gain benefit from the experience of other, possibly larger and longer established and more experienced patient organisations, some member associations collaborated with others nationally and internationally. Umbrella organisations, such as the EGA and those for other rare diseases, were also a resource of advice, expertise and support.

Increasing membership of patient associations can help to drive activities in supporting patients and families but three member organisations expressed concern over the few patients and families joining patient organisations in their countries and/or their reluctance to become involved in activities. This, as suggested by the Belgian respondent, may be partly because most patients have access to treatment, feel well, and do not perceive a need to belong to a patient organisation. This, however, ignores the on-going difficulties of some patients, wherever in the world they are located, that do not have adequate treatment available to them (patients with neuronopathic disease or patients who cannot gain funding for treatment) and those who do not have access to adequate healthcare services.

## Conclusions

Although there are several limitations in the conduct of this survey in that not all patient organisations answered all questions, different patient organisations may have interpreted question differently and provided varying levels of detail or approximations in their responses, the survey revealed some insights into the priorities and needs of Gaucher patient organisations and of the patients they represent. A key priority is to work towards equitable access to treatment and equitable standards of healthcare. Another important and related need is to increase awareness of Gaucher disease, its manifestations and impact on patients’ lives, amongst the medical community and amongst reimbursement agencies. Of growing concern is the worsening economic climate in many countries, which creates unease for the future funding of treatments for Gaucher disease and other rare diseases, and also for the future maintenance of patient organisations’ activities.
